# Fifteen-Year-old Girl With Fever, Headache and Neck Stiffness

**DOI:** 10.1097/INF.0000000000004532

**Published:** 2024-09-17

**Authors:** Katerina Gramm, Tram Pham Ngoc, Le Huu Dang Nhat, Vu Thi Thuy Duong, Tran Ngoc Luu, Julie Huynh

**Affiliations:** From the * Medical Sciences Division, University of Oxford, Oxford, United Kingdom; † Oxford University Clinical Research Unit, Ho Chi Minh City, Vietnam; ‡ Children’s Hospital 2, Ho Chi Minh City, Vietnam; § Nuffield Department of Medicine, Centre for Tropical Medicine and Global Health, University of Oxford, Oxford, United Kingdom.

**Keywords:** meningitis, *Streptococcus suis*, pediatrics, case report

## CASE

A 15-year-old female from rural Vietnam was transferred from a local hospital to a pediatric tertiary referral center in Ho Chi Minh City, with 3 days of fever and headache. On presentation, she had reduced consciousness (Glasgow Coma Scale score of 11—eye response 2, voice response 3 and motor response 6) and was significantly agitated. The patient weighed 49 kg and was lethargic with high fevers, up to 39.5 °C. Other vital signs were heart rate 100 beats/min, blood pressure 130/80 mm Hg, respiratory rate 20 breaths/min and oxygen saturations 100% on room air.

On neurologic examination, she had nuchal rigidity, and Kernig’s and Brudzinski’s signs were not formally tested. There was no focal neurology, including no weakness or sensory changes. Tone, reflexes and coordination were normal. Cardiorespiratory and abdominal examinations were normal and no skin rashes were present. Examination of lymph nodes was normal.

Her past medical history included a solid pseudopapillary tumor of the pancreas with metastases to her spleen, for which she had surgical resection of the body and tail of the pancreas and a total splenectomy 3 months before admission. She had not been receiving any postsplenectomy antibiotic prophylaxis or additional vaccinations. She recovered well without complications and surgical scars were healing well.

The patient was vaccinated as per the national childhood vaccination program in Vietnam. She had no sexual or social history which put her at risk of HIV transmission. There was no recent tuberculosis exposure nor recent travel outside of Vietnam or direct contact with farm or domestic animals.

Full blood count showed hemoglobin 12.2 g/dL, white blood cell count 26.12 × 10^9^/L (91.2% neutrophils, 3.8% lymphocytes, 3.9% monocytes, 0.9% eosinophils and 0.2% basophils), platelets 271 × 10^9^/L, CRP 154.2 mg/L and blood glucose 7.1 mmol/L. Lumbar puncture was performed, which showed 2363 leukocytes/µL with 94% polymorphonuclear cells and 6% mononuclear cells, 1000 red blood cells/µL, protein 1.97 g/L, glucose 1.8 mmol/L and lactate 10.1 mmol/L in the cerebrospinal fluid (CSF). Empirical intravenous (IV) ceftriaxone 4 g daily and vancomycin 3.6 g daily were commenced. Raised intracranial pressure characterized by lethargy and hypertension was managed with mannitol. After 1 hour of growth, CSF grew Gram-positive cocci. Due to persistent fevers, a repeat lumbar puncture was performed on day 12 of admission. CSF results showed white blood cells 24 cells/µL (100% mononuclear cells), nil red blood cells, protein 0.18 g/L, glucose 4.1 mmol/L and lactate 2.0 mmol/L. A computed tomography scan of the brain, without contrast, was normal without evidence of hydrocephalus, identifiable intracranial abscesses or hemorrhage. A chest radiograph and subsequent ultrasound scan of the lungs showed small bilateral pleural effusions, which were more prominent on the right.

The patient improved clinically and became more alert and eventually defervesced. She continued to have a headache, although it had improved markedly.

## DENOUEMENT

Blood and CSF cultures returned positive for *Streptococcus suis* susceptible to penicillins (minimum inhibitory concentration for penicillin ≤0.06), cephalosporins, including ceftriaxone (minimum inhibitory concentration 0.25) and vancomycin (minimum inhibitory concentration 0.25). IV ceftriaxone and vancomycin were continued. After 6 days of treatment, repeat blood culture continued to be positive for *S. suis*, however, repeat CSF was sterile. To investigate possible persistent sources causing the patient’s delayed clearance of the organism, cardiac ultrasound and computed tomography scan of the brain, with contrast, were carried out, both of which were normal. After 17 days of treatment, blood cultures were negative. The patient developed an acute kidney injury during admission, likely secondary to vancomycin therapy (urea peaked at 5.4 mmol/L and creatinine at 261 µmol/L). IV vancomycin was substituted with IV linezolid, and the acute kidney injury resolved. The patient was discharged after a total of 24 days of antimicrobial therapy. She was discharged without any obvious neurologic sequelae, and her hearing acuity was normal on formal audiology. At 1-month follow-up, the creatinine had normalized.

*S. suis* is a Gram-positive facultative anaerobe, with 29 serotypes currently recognised.^1^ Serotype 2 is considered to be the most virulent and most commonly causes disease in humans.^2^ Pigs are the natural reservoir of *S. suis*, which has been isolated worldwide as a commensal in the intestinal flora.^3^ Most cases of *S. suis* infection have been reported in South East Asia, where there is high density of pig populations.^4^ The most common clinical manifestation of *S. suis* infection in adults is meningitis, although other clinical syndromes include sepsis, arthritis and endocarditis.^5^ In the south of Vietnam, *S. suis* serotype 2 is the most common causative pathogen of meningitis in adults.^5,6^ *S. suis* meningitis has a low mortality rate of 2.9% in adults, although there is substantial risk of hearing loss.^7^ We were unable to ascertain the serotype that affected the patient in this case, as serotyping was not available.

The mean age of patients with *S. suis* meningitis is 49 years. Occupational exposure to pigs has been reported as risk factor for *S. suis* infection, leading to reasoning that this is mainly a disease of working adults, who are usually infected via skin lesions.^7,8^ Indeed, the mean age of *S. suis* meningitis patients is significantly higher than other causes of bacterial meningitis.^8^ Despite the well-known role of *S. suis* as a cause of meningitis in adults, there are few reports describing *S. suis* meningitis in children in South East Asia, and the mortality and morbidity of this condition in children remain unclear.^9^

After the cultures returned positive for *S. suis*, staff enquired about risk factors for this infection, namely prior exposure to pigs. The patient and her family initially denied any exposure to pig, but admitted that 3 days before her symptoms began, she ate pork organ congee, or Cháo Lòng, which she had bought from a street food vendor in her local area. This is a traditional Vietnamese dish based on savory rice porridge, with the addition of pork offal, including pig intestine. The patient likely contracted *S. suis* from eating this undercooked pork congee. The incubation period in this case was 3 days, consistent with incubation periods reported in the literature.^9^ Eating raw pork has been shown to be a risk factor for *S. suis* meningitis in adults, leading to infection via the gastrointestinal tract.^7,10^ *S. suis* serotype 2 is able to translocate across human intestinal epithelial cells, explaining its potential to infect humans as a food-borne pathogen.^10^

The patient had undergone a splenectomy, which may have predisposed to *S. suis* infection, especially as *S. suis* is an encapsulated organism, which splenectomized patients are vulnerable to.^9,11^ For other *Streptococcus* species, in particular S. *pneumoniae*, splenectomy is well-reported as a risk factor for severe infection.^11^ However, the literature remains more unclear in regard to *S. suis* infection, specifically *S. suis* meningitis, as few patients included in published reports had undergone a splenectomy.^6,8^ However, splenectomy leading to immunocompromise was likely the main contributor to the patient’s delayed clearance of *S. suis*. The patient was not discharged with regular antibiotic prophylaxis nor received any immunizations, as this is not common practice in Vietnam owing to social and financial challenges. This is in contrast to guidance followed by most Western countries, where daily prophylaxis with penicillin-based antibiotics is recommended for a minimum of 2 years postsplenectomy, and vaccination against encapsulated bacteria is recommended peri-splenectomy.^11^

This patient was treated with IV ceftriaxone and vancomycin, to which *S. suis* was susceptible. Ceftriaxone with the optional addition of vancomycin has been recommended previously as empirical treatment, in accordance with local guidelines for bacterial meningitis, which should ideally be based on local epidemiologic data of the causative pathogens.^4^ Although not present in this case, multidrug resistance in *S. suis* is becoming an increasingly prevalent issue. In the south of Vietnam, serotyping has proven that resistance has increased over the period of more than a decade studied.^12^ The majority (83.2%) of *S. suis* samples isolated in a Vietnamese study were resistant to tetracycline, and a significant minority (20.2%) were also resistant to erythromycin. However, all samples in this study were susceptible to penicillin, ceftriaxone and vancomycin.^6^ This underlines the importance of antimicrobial stewardship, and empirical antibiotic choice should be replaced promptly with more focused narrow-spectrum antibiotics, such as penicillin, once antimicrobial susceptibilities have been confirmed.

The role of dexamethasone in treating *S. suis* meningitis has been controversial; a randomized controlled trial in Vietnam found that dexamethasone reduced hearing loss in adults with *S. suis* meningitis to 12% versus 38% with placebo, but there was no significant reduction in mortality.^13^ However, a Cochrane review found no reduction in hearing loss in children with non-*Haemophilus* species meningitis, including for *Streptococcus pneumoniae* meningitis, although the review did not include studies in which children were affected by *S. suis* meningitis, due to the paucity of data in this population.^14^

Measures to prevent the spread of infection are critical, including improved hygiene measures within the food industry, such as slaughterhouses, processing centers and food manufacturers.^2^ Improved food safety education is also necessary for the wider population, including street vendors, to minimize the risk of undercooked pork being served to customers. Simple food safety measures include hand washing after handling raw meat, and ensuring that meat is cooked thoroughly before being eaten.^8^ However, there are traditional dishes in which raw pork is a delicacy. For example, Tiết Canh is a Vietnamese dish consisting of raw pork blood pudding, which is more frequently consumed by rural populations and has been linked to *S. suis* infection.^15^ Hence, it may be challenging to thoroughly eradicate *S. suis* infection transmitted through undercooked or raw animal products, and public health initiatives should focus on educating populations, especially rural, on the dangers of consuming these dishes and the importance of food hygiene.
